# Skeletal Muscle Angiopoietin-Like Protein 4 and Glucose Metabolism in Older Adults after Exercise and Weight Loss

**DOI:** 10.3390/metabo10090354

**Published:** 2020-08-31

**Authors:** Guoyan Li, Hefang Zhang, Alice S. Ryan

**Affiliations:** 1 Department of Medicine, Division of Gerontology and Geriatric Medicine, University of Maryland School of Medicine, Baltimore, MD 21201, USA; guoyanli@som.umaryland.edu (G.L.); hefang.zhang@outlook.com (H.Z.); 2 VA Research Service, Baltimore VA Medical Center, Baltimore, MD 21201, USA; 3 Baltimore Geriatric Research, Education and Clinical Center (GRECC), VA Maryland Health Care System, Baltimore, MD 21201, USA

**Keywords:** exercise, skeletal muscle, glucose metabolism, lipids, obesity

## Abstract

Angiopoietin-like protein 4 (ANGPTL4) is an adipokine that plays an important role in energy homoeostasis and lipid and lipoprotein metabolism. This study was designed to determine the effect of an exercise plus weight loss intervention on ANGPTL4 expression and its relationship with metabolic health. Thirty-five obese sedentary men (*n* = 18) and postmenopausal women (*n* = 17), (X *±* SEM, age: 61 *±* 1 years, BMI: 31.3 *±* 0.7 kg/m^2^, VO_2_max: 21.7 *±* 0.9 L/kg/min) completed a 6 month program of 3×/week aerobic exercise and 1×/week dietary instruction to induce weight loss (AEX + WL). Participants underwent vastus lateralis muscle biopsies, a hyperinsulinemic–euglycemic clamp, oral glucose tolerance tests and body composition testing. Basal skeletal muscle ANGPTL4 mRNA was lower in men than women (*p* < 0.01). Peroxisome proliferator-activated receptor (PPAR) alpha (PPARα) mRNA expression was higher in men than women (*p* < 0.05). There were no significance changes in serum or skeletal muscle ANGPTL4 (basal or insulin-stimulated) or muscle PPARα mRNA expression after AEX + WL. Muscle mRNA ANGPTL4 is correlated with serum ANGPTL4 (*r* = 0.41, *p* < 0.05), body fat (*r* = 0.64, *p* < 0.0001), and glucose utilization (*r* = 0.38, *p* < 0.05). AEX + WL does not change basal or insulin-stimulated skeletal muscle ANGPTL4 mRNA expression, suggesting other factors contribute to improved insulin sensitivity after the loss of body fat and improved fitness.

## 1. Introduction

Angiopoietin-like protein 4 (ANGPTL4), a member of the angiogenin-like protein family, is expressed in a wide variety of tissues, such as liver, adipose tissue, skeletal muscle, placenta, small intestine, brain, thyroid, kidney, spleen, pituitary gland, hypothalamus and heart [1,2,3,4,5,6,7,8], while predominately expressed in the liver, adipose tissue and placenta [8,9,10] and induced in multitude of physiological conditions, such as fasting, hypoxic environments, pregnancy and lactation, adipocyte differentiation and exercise [1,3,4,11]. There is evidence that ANGPTL4 is directly related to the risk of type 2 diabetes and insulin resistance in an animal model [12]. ANGPTL4 serum levels are significantly higher in adults with abnormal glucose tolerance and are associated with central and total obesity and insulin resistance by the Homeostatic Model Assessment of Insulin Resistance (HOMA IR) [13]. In contrast, an earlier study reported that serum ANGPTL4 levels are significantly lower in patients with type 2 diabetes mellitus compared with healthy adults [12] and circulating ANGPTL4 levels were negatively associated with plasma glucose and insulin resistance by HOMA IR in humans [12]. One study showed that serum ANGPTL4 was reduced by 2 h insulin infusion during a two-step hyperinsulinemic clamp [14]. Although serum or plasma ANGPTL4 levels are related to obesity and insulin resistance, its expression in skeletal muscle specifically during basal conditions and hyperinsulinemia, and its relationship with insulin sensitivity, have not been well studied in older, generally healthy, adults, nor compared between men and women. 

It is possible that ANGPTL4 could be modified by exercise or diet as regular exercise training is an effective stimulant of muscle metabolism remodeling. Immediately after an acute bout of endurance and strength exercise, both serum and skeletal muscle ANGPTL4 mRNA increase in healthy, sedentary middle-aged men [3]. Similar findings are observed in animal models where 50 min of running significantly increases ANGPTL4 protein and mRNA expression levels in gastrocnemius and soleus muscles in mice [15]. ANGPTL4 is released mainly from the liver in response to exercise in animals [5]. It has been shown that the induction of ANGPTL4 in non-exercising muscle is mediated by elevated plasma FFA via PPARδ, presumably leading to preventing fat overload and providing fatty acids to the active skeletal muscle [4]. Additionally, Ingerslev and colleagues [5] have shown that ANGPTL4 is mainly released from the liver in response to exercise. An increase in body weight increases serum ANGPTL4 levels, whereas a loss of body weight decreases ANGPTL4 serum levels in young and middle-aged adults [13]. Yet, little is known regarding the effects of exercise training combined with weight loss in older adults on ANGPTL4 expression levels in skeletal muscle and in serum.

ANGPTL4 is the target of peroxisome proliferator-activated receptors (PPAR) and induced by PPARα in mice and rat models [16,17,18]. Muscle-derived ANGPTL4 is induced by fatty acids via PPARδ, but not PPARα, and PPARγ in human skeletal muscle myotubes [19]. PPARα plays a major role in regulating fatty acid (FA) transport and β-oxidation [20]. The activation of PPARα can improve hepatic insulin resistance by reducing the accumulation of deleterious lipids [21]. As ANGPTL4 regulates lipid metabolism by inhibiting lipoprotein lipase activity and stimulating lipolysis in white adipose tissue, it may play a role mediating insulin resistance through the regulation of its expression by PPARs (PPARα, PPARβ/δ, PPARγ) [8]. Therefore, the aim of this investigation was to determine the effects of a 6-month aerobic training + weight loss program on ANGPPTL4 and PPARα expression in skeletal muscle and their relationship with insulin resistance in older obese adults and to examine differences in ANGPTL4 expression between men and women.

## 2. Results

### 2.1. Effects of Exercise Plus Weight Loss

The baseline physical and metabolic characteristics and gene expressions of the subjects (*n* = 35) before and after the intervention are presented in Table 1. Body weight decreased 8% (*p* < 0.001) with a decrease in the percentage of body fat of 11% (*p* < 0.001), a 17% decrease in total fat mass (*p* < 0.001), a 20% decrease in visceral adipose tissue (VAT), a 16% decrease in subcutaneous abdominal fat and a 12% decrease in mid-thigh subcutaneous fat (*p* < 0.001). There was no change in fat-free mass (FFM) or mid-thigh muscle area (Table 1). Muscle attenuation increased (*p* < 0.05). VO_2_max (mL/kg//min) increased 21% after aerobic exercise + weight loss (AEX + WL) (*p* < 0.001).

**Figure 1:**
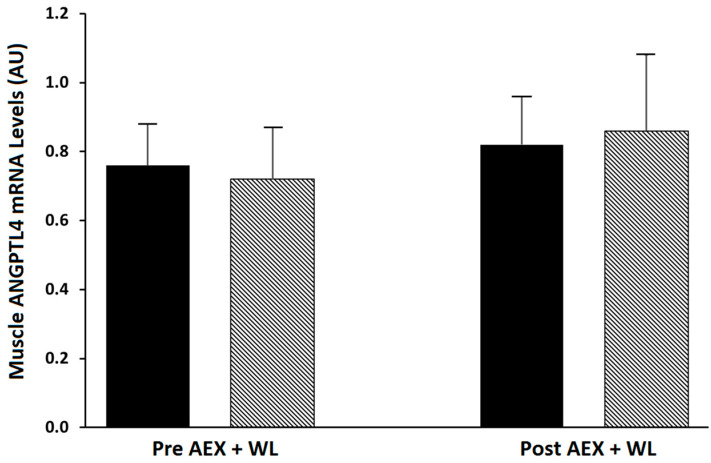
Skeletal muscle angiopoietin-like protein 4 (ANGPTL4) levels during basal (solid) and insulin-stimulated (striped) conditions pre aerobic exercise + weight loss (AEX + WL) and post AEX + WL (*n* = 35).

At baseline, 20 subjects had normal glucose tolerance, 13 had impaired glucose tolerance, and 2 had unconfirmed diabetes [22]. Fasting plasma glucose concentrations did not change after AEX + WL but fasting insulin levels were reduced (*p* < 0.001, Table 1). Glucose AUC120 and glucose AUC180 during the oral glucose tolerance test (OGTT) decreased 6 and 5%, respectively (*p* < 0.05) and insulin AUC120 and insulin AUC180 during the OGTT both decreased 17% (*p* < 0.01). Glucose utilization and insulin sensitivity increased between 18 and 40% (all *p* < 0.0001). There were no significant changes in skeletal muscle ANGPTL4 mRNA, PPARα mRNA and serum ANGPTL4 protein level after AEX + WL (Table 1). There was no significant change in basal vs. insulin-stimulated ANGPTL4 mRNA either before (0.759 *±* 0.17 vs. 0.724 *±* 0.150 AU) or after (0.817 *±* 0.139 vs. 0.858 *±* 0.218 AU) AEX + WL (Figure 1). There were no significant changes in muscle ANGPTL4 mRNA levels with AEX + WL by glucose tolerance status (NGT, *n* = 20: 0.74 *±* 0.16 vs. 0.73 *±* 0.17 AU or IGT, *n* = 15: 0.95 *±* 0.17 vs. 0.72 *±* 025 AU).

### 2.2. Comparisons between Men and Women

**Figure 2:**
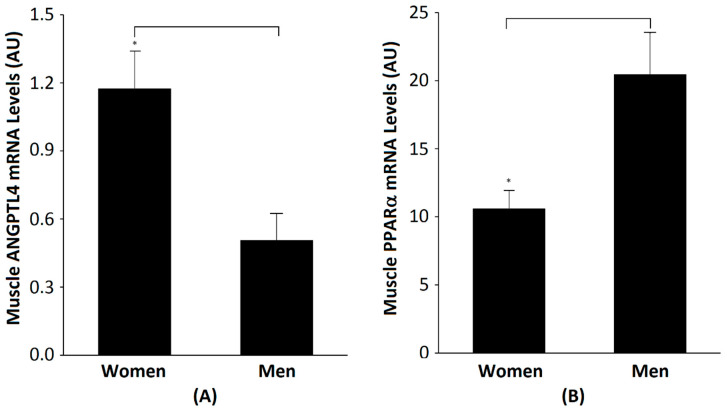
(**A**) Relative skeletal muscle ANGPLT4 mRNA expression at baseline (pre AEX + WL) in women (*n* = 17) and men (*n* = 18) (*p* ˂ 0.01, between groups) and (**B**) relative skeletal muscle PPARα mRNA expression at in women (*n* = 8) and men (*n* = 9), (* *p* < 0.05, between groups).

Baseline physical and metabolic characteristics, and the gene expressions of men (*n* = 18) and women (*n* = 17), are presented in Table 2. The men and women had comparable BMIs and fat masses. Men had higher body weight, FFM and VO_2_max than women (all *p* < 0.05). The men had a significantly lower percent of body fat than women. Women had higher total cholesterol, HDL-cholesterol and LDL-cholesterol than the men (*p* < 0.05). Fasting glucose, insulin and serum ANGPTL4 were not significantly different between groups. Skeletal muscle ANGPTL4 mRNA was lower in men than women (*p* ˂ 0.01) (Figure 2A). There are significant differences between men and women regardless of glucose tolerance status (NGT men (*n* = 10) vs. NGT women (*n* = 10): 0.14 *±* 0.19 vs. 1.07 *±* 0.22, *p* < 0.05) and (IGT men (*n* = 8) vs. IGT women (*n* = 7): 0.63 *±* 0.13 vs. 1.33 *±* 0.26, *p* < 0.05). PPARα mRNA was higher in men than in women (*p* ˂ 0.01) (Figure 2B). There was no significant change in skeletal muscle ANGPTL4 after AEX + WL in men (0.507 *±* 0.119 vs. 0.493 *±* 0.095 AU) or women (1.174 *±* 0.167 vs. 1.307 *±* 0.244 AU). In addition, there were no significant changes in serum ANGPTL4 in men (343.8 *±* 48.0 vs. 372.3 *±* 70.4 pg/mL) or women (374.3 *±* 71.4 vs. 450.5 *±* 94.4 pg/mL).

### 2.3. Correlations with ANGPTL4 mRNA

**Figure 3:**
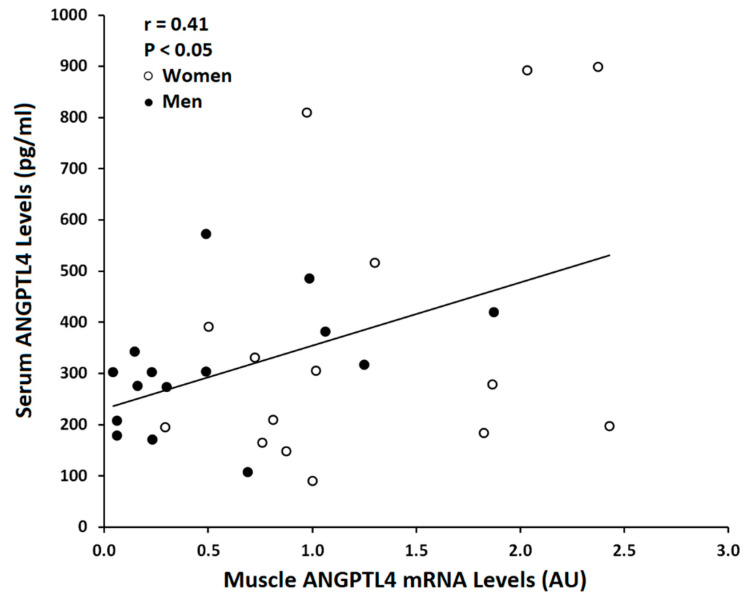
Relationship between muscle ANGPTL4 mRNA level and serum ANGPTL4 level (Total: *n* = 30, Women ○: *n* = 15, Men ●: *n* = 15; r = 0.41, *p* < 0.05) before the intervention.

**Figure 4:**
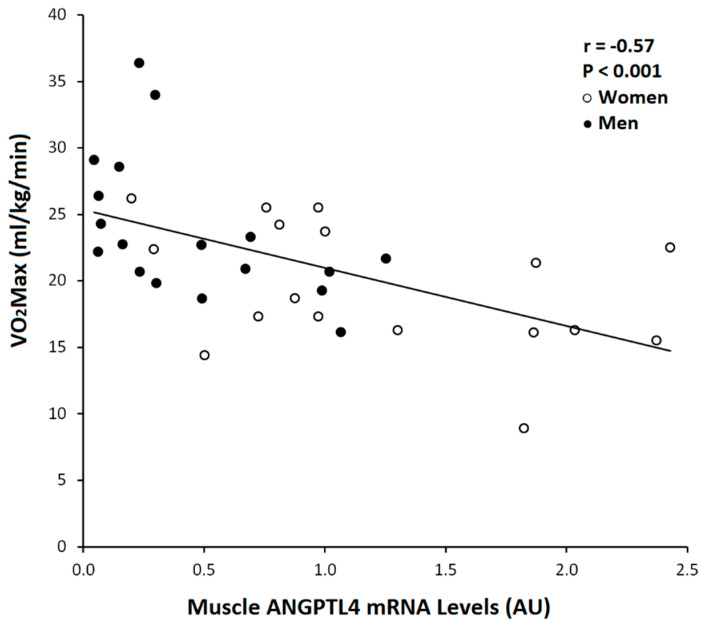
Relationship between muscle ANGPTL4 mRNA level and VO_2_max (Total: *n* = 35, Women ○: *n* = 17, Men ●: *n* = 18; r = 0.57, *p* < 0.001) at baseline.

**Figure 5:**
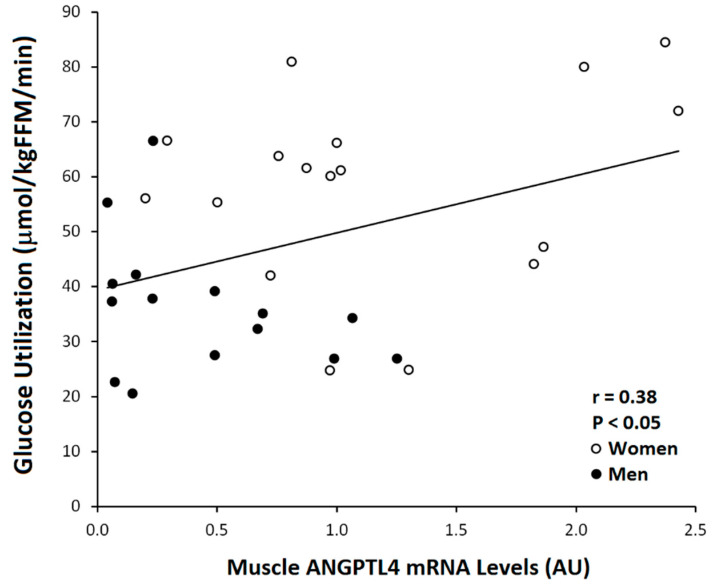
Relationship between muscle ANGPTL4 mRNA level and glucose utilization (M) (Total: *n* = 32, Women ○: *n* = 17, Men ●: *n* = 15; r = 0.38, *p* < 0.05) at baseline.

Skeletal muscle ANGPTL4 mRNA was associated with serum ANGPTL4 in the total group (r = 0.41, *p* < 0.05, Figure 3). Muscle ANGPTL4 was inversely correlated with VO_2_max (r = −0.50, *p* < 0.005; Figure 4). Basal ANGPTL4 mRNA levels were positively associated with percent fat, fat mass, mid-thigh fat and total cholesterol, HDL and LDL-cholesterol (Table 3). Basal ANGPTL4 mRNA levels were negatively correlated with mid-thigh muscle area and FFM. Basal PPARα mRNA level was negatively associated HDL (r = −0.55, *p* = 0.027). Muscle ANGPTL4 was associated with M, only when expressed per FFM (Figure 5), prior to the intervention (r = 0.38, *p* < 0.05, Table 3), but was not associated with insulin sensitivity (M/I). There were no associations between BMI, abdominal fat, TG and skeletal muscle ANGPTL4 gene expression (data not shown). Skeletal muscle ANGPTL4 tended to be associated with muscle PPARα mRNA in men (r = 0.62, *p* = 0.08) and women (r = 0.68, *p* = 0.096). Serum ANGPTL4 was associated with BMI (r = 0.46, *p* < 0.05), percent body fat (r = 0.38, *p* < 0.05) and fat mass (r = 0.46, *p* < 0.05).

## 3. Discussion

This study showed higher skeletal muscle ANGPTL4 levels in women than men and that a six-month aerobic exercise weight loss program did not change basal and insulin-stimulated ANGPTL4 mRNA expression, despite successful improvements in fitness, glucose tolerance and insulin sensitivity and reductions in body weight and abdominal fat.

Our study aimed to investigate whether aerobic training combined with weight loss could alter the muscle and serum levels of ANGPLT4. Muscle ANGPLT4 did not change in the total group or in either men or women alone. ANGPLT4 may be an exercise responsive myokine. An acute bout of exercise in mice results in the upregulation of skeletal muscle ANGPTL4 [15]. Norheim et al. reported that, immediately after and 2 h post exercise at baseline and after the completion of a 12-week training period, serum ANGPTL4 level and skeletal muscle ANGPTL4 mRNA increased during acute exercise and remained higher after 2 h [3]. The effect of this increase is more pronounced in normal glycemic adults than overweight dysglycemic adults with the opposite direction in serum suggesting that other tissues, possibly adipose tissue and liver, contribute to circulating effects. In the same study, 12-weeks of exercise training did not change the serum ANGPTL4 level and skeletal muscle ANGPTL4 mRNA [3]. Catoire et al. [4] reported that, during acute exercise in men, the expression of ANGPLT4 is elevated in non-exercising muscle, leading to the reduced local uptake of plasma triglyceride-derived fatty acids and their sparing for use by exercising muscle. One study, conducted in young obese adults, demonstrated an increase in serum ANGPTL4 after both 12 weeks of exercise alone or diet alone, but did not show any change in serum ANGPTL4 after the combined diet and exercise program [23]. In contrast, ANGPTL-4 mRNA in adipose tissue decreased after exercise, diet, and diet and exercise, indicating that circulating factors may respond differently than tissue sources. Our findings that a 6-month AEX + WL intervention does not change the fasting levels of serum ANGPTL4 in the total group or in men or women alone is consistent with the study in young adults and overweight/obese participants [23] and in a study of weight loss alone [24]. Since sample sizes were similar (~19 per group [23]) or lower (9 per group [24] ) than ours, reasons for the disparity in results in terms of changes in ANGPTL-4 are unknown but could be partly explained by age (young vs. middle-aged and older), BMI and type of tissue (adipose tissue vs. skeletal muscle). Furthermore, the results suggest that ANGPTL4 affects the short-term redistribution of energy utilization but does not appear to have a long-term effect.

We also examined the associations between muscle ANGPTL4 and obesity, aerobic capacity, glucose tolerance and insulin sensitivity. Our results are in agreement with studies that demonstrate that ANGPTL-4 levels are positively correlated to BMI, abdominal obesity (by waist circumference) and fat mass [13]. Our observed relationships between muscle ANGPTL4 and measures of obesity are novel, although others have reported relationships with serum ANGPTL4 and total body fat mass [13]. Differences in glucose metabolism (glucose utilization or M by the clamp), obesity (fat mass), and fitness (VO_2_max) between men and women could explain the sex differences in the regulation of muscle ANGPLT4 levels. ANGPTL4 serum levels are significantly higher in obese patients with abnormal glucose tolerance and positively correlated with BMI, waist circumference, fat mass, HbA1c, HOMA-IR, fasting triglycerides, and with inflammatory markers [13]. Although our results demonstrate a positive relationship between muscle ANGPTL4 and glucose utilization (when expressed per FFM), this may be driven by both the difference in insulin sensitivity between men and women and differences in ANGPTL4 between men and women. We did not find a relationship between serum levels and glucose utilization.

In young and middle-aged adults, serum levels of ANGPTL4 were inversely related to fasting glucose, and HOMA-IR levels were lower in patients with type 2 diabetes than healthy adults [12]. Using an adenovirus-mediated expression system in mice, Xu et al. [12] reported that ANGPTL4 markedly improved glucose tolerance and decreased blood glucose, possibly by the inhibition of hepatic glucose production. Further, oil Red O staining showed that lipid droplets accumulated diffusely in the liver of Adv-ANGPTL4-treated mice, suggesting that ANGPTL4 overexpression induces a fatty liver [12]. ANGPTL4 treatment activates AMP-activated protein (AMPK) signaling and mitochondrial oxidative capacity in skeletal muscle [15] and mice deficient in ANGPTL4 have a lower exercise endurance capacity [15]. This might suggest that muscle ANGPTL4 would be directly related to VO_2_max, in contrast to our findings. We are unaware of any studies that have examined the relationship between muscle ANGPTL4 and VO_2_max. Since all the participants in our study were sedentary at baseline, the variability in fitness levels was limited. It is possible that including a wider range of VO_2_max might result in different findings.

Our data show that the baseline levels of ANGPTL4 and PPARα are positively correlated in both genders. However, the expression of ANGPTL4 in skeletal muscle is much higher in women than in men, while the expression of PPARα is the opposite, implying that PPARα is more efficient (requires a lower concentration) in regulating the expression of ANGPLT4 in women than in men. According to other studies [1,25], a PPARα agonist can increase cleaved ANGPTL4 in human plasma. In a mouse model, it can stimulate ANGPTL4 expression in the liver and kidney, but decreases ANGPTL4 expression in mouse heart, skeletal muscle and adipose tissue. Although these studies utilize animal models, this may explain the higher skeletal muscle PPARα but lower AGNPTL4 in men, and the lower expression of PPARα but higher ANGPTL4 expression in skeletal muscle in women. Peters et al. reported that, in a mouse model, lacking PPARα function results in higher levels of serum HDL [26]. Although we were able to measure PPARα in only a limited number of samples in our study, muscle PPARα mRNA was negatively correlated with HDL. On the other hand, ANGPTL4 is positively associated with plasma cholesterol, HDL-C, LDL-C and intramuscular fat. Thus, both PPARα and ANGPTL4 impact circulating lipids level.

The study limitations include the small sample size for the measurement of skeletal muscle PPARα and the absence of measurements of PPARδ and PPARγ which could be examined in future studies. Results are generalizable to generally healthy older mostly Caucasian adults and would need to be confirmed in other racial and ethnic groups. However, we conducted a rigorous exercise and weight loss program and employed the gold-standard glucose clamp and had sophisticated methods to measure fitness and body fat. Furthermore, we provided food for two days prior to the glucose clamp and the muscle biopsy; thus, controlling for any immediate effect of dietary intake. This is important given that meal fatty acid composition affects plasma ANGPTL4, as ANGPTL4 is secreted from human muscle after a high-saturated fatty acid mixed meal [27], but plasma levels of ANGPTL4 are not associated with skeletal muscle LPL activity after the meal. The fasting conditions in our study perhaps allowed us to find a significant relationship between serum ANGPTL4 level and skeletal muscle ANGPTL4 mRNA level.

## 4. Materials and Methods

### 4.1. Participants

Study entry criteria were men and post-menopausal women 50–80 years of age who were nonsmokers and had no previous diagnoses of diabetes or cardiovascular disease. The participants also had to be weight stable (<2.0 kg weight change in the past year), overweight and obese (body mass index, BMI >25 kg/m^2^; range of 25–46 kg/m^2^) and sedentary (<20 min of aerobic exercise 2×/week). They were between the ages of 54–77 years, of whom 27 were Caucasian and 8 were African–American. All subjects were nonsmokers, showed no evidence of cancer, liver, renal or hematological disease, or other medical disorders by medical history and physical exam. A graded exercise treadmill test excluded those with asymptomatic coronary artery disease. Individuals with untreated hypertension (blood pressure higher than 160/90 mm Hg) or hyperlipidemia (triglycerides > 4.5 mM, cholesterol > 6.2 mM, LDL cholesterol > 4.3 mM) were referred to their doctor for therapy. The women in the study were post-menopausal and had not menstruated for at least 1 year. Participants (*n* = 35) met all the study criteria (18 men and 17 women). Samples from the men were from a study of genetics and insulin sensitivity. Samples from the women were a subset of a prior investigation in our lab [28] (Clinical Trials: NCT00882141). The final study included those who had samples available for analyses. The Institutional Review Board of the University of Maryland approved all methods and procedures. Each participant provided written informed consent to participate in the study.

### 4.2. Exercise and Weight Loss Intervention

The research was conducted at the University of Maryland Baltimore, and Baltimore Veteran Affairs Medical Center from 2001–2008, and lab analysis in 2018–2019. Adults were enrolled in a dietary induced weight loss plus moderate-to-high intensity aerobic exercise intervention (AEX + WL) for 6 months. Before beginning the weight-loss intervention, all subjects completed an initial 7-day food record and then received instructions for maintaining stable weight, through a Therapeutic Lifestyle Changes (TLC) diet [29], from a registered dietitian (RD) one day/week for 6–8 weeks. All participants attended weekly weight loss classes led by an RD for instruction in the TCL diet. Compliance, defined as meeting the TLC recommendations, was monitored by 7-day food records (or 24-h recalls) using the American Diabetes Association exchange list system. Individuals were instructed to restrict their caloric intake by 500 kcal/day. All participants completed three aerobic exercise sessions a week, initially exercising on a treadmill at ~50–60% VO_2_max for 20–30 continuous minutes, as tolerated. After 3–4 weeks, time was progressed to 50 min and then the duration remained at 50 min for the remainder of the intervention. The intensity progressed to >60% VO_2_max and up to 80% VO_2_max. This progression of intensity typically took 4–6 weeks based on each subject’s exercise tolerance. Exercise intensity, prescribed as a target heart rate range, was monitored using chest-strap heart rate monitors (Polar Electro Inc., Lake Success, NY). All sessions were supervised by exercise physiologists. Exercise sessions included a warm-up and a cool-down phase for each of 5–10 min. The average compliance, defined as attending each of the exercise sessions and weight loss classes was greater than 75%.

### 4.3. Outcome Measures

#### 4.3.1. VO_2_max and Body Composition

VO_2_max was measured using a continuous treadmill test protocol, as previously described [30]. Height (cm) and weight (kg) were measured to calculate body mass index (BMI) and waist and hip circumferences to obtain waist-to-hip ratio (WHR). One participant did not undergo the computed tomography (CT) scan at either timepoint and five missed the CT scan after AEX + WL. Percent body fat, fat mass, lean tissue mass and bone mineral content (BMC) (fat-free mass= lean + BMC) were determined by dual-energy X-ray absorptiometry (DXA) (Prodigy, LUNAR Radiation Corp., Madison, WI). A single computed tomography (CT) scan at L4–L5 region using a Siemens Somatom Sensation 64 Scanner (Fairfield, CT) was used to determine visceral adipose tissue (VAT) and subcutaneous adipose tissue (SAT) areas and analyzed using MIPAV (NIH Image Analysis Program). Muscle area, total fat area, and low-density lean tissue area/intramuscular fat (IMAT) was determined in a second scan of the right leg [30].

#### 4.3.2. Oral Glucose Tolerance Test (OGTT) and Lipids

All participants were weight stabilized (*±* 2%) for at least two weeks prior to testing before and after the AEX + WL and were provided with a eucaloric diet for two days before the clamp by an RD to control nutrient intake [30]. All testing was performed in the morning after a 12-h overnight fast. One man did not undergo the OGTT test. At the end of the 6-month program, subjects were asked to continue the aerobic training 3 days/week during the final testing period and the glucose clamps were performed 36–48 h after the last bout of exercise.

Blood samples were drawn before and at 30-min intervals for 3 h after the ingestion of 75 g of glucose. Plasma glucose concentrations were measured using the glucose oxidase method (2300 STAT Plus, YSI, Yellow Springs, OH). Plasma insulin was measured by RIA (Linco Research, St. Charles, MO). Glucose and insulin total area under the curve at 120 and 180 min (GlucoseAUC and InsulinAUC, respectively) were calculated by the trapezoidal method. The lipid levels were obtained from three separate fasting visits and the results averaged as previously described [31].

#### 4.3.3. Glucose Clamp and Skeletal Muscle Biopsies

Testing was conducted after the participants underwent a 12-h fast. Peripheral tissue sensitivity to exogenous insulin (M) was measured using the hyperinsulinemic–euglycemic clamp technique [32] for 180 min at 80 mU.m^−2^.min^−1^ (Humulin, Eli Lilly Co., Indianapolis, IN) [33]. Three men did not undergo the glucose clamp. Insulin-stimulated glucose uptake per unit plasma insulin (M/I, an index of insulin sensitivity), which represents the amount of glucose metabolized per unit of plasma insulin (I), was calculated by diving the glucose utilized by the insulin concentration during the last 60 min of the clamp for each individual. Vastus lateralis percutaneous needle muscle biopsies were taken from each participant under local anesthesia using a 5 mm Bergstrom needle (Stille, Solna, Sweden). Muscle was immediately freeze-clamped and stored at −80°C. Approximately 50–80 mg of muscle was used for RNA isolation and ANGLT and PPARα gene expression. Total RNA extraction, cDNA synthesis and Quantitative Real-Time PCR (qPCR) were done by our standard laboratory methods [33]. The Primer/Probes were from ThermoFisher—ANGLT4: assay ID Hs01101127_m1; PPARα: assay ID Hs00231882_m1. The expression levels of each mRNA were normalized to 36B4 mRNA.

#### 4.3.4. Serum ANGPTL4

ANGPTL4 serum level was measured in duplicate by enzyme-linked immunosorbent assay (ELISA) (Human ANGPTL4 ELISA kit. Invitrogen, Cat#: EHANGPTL4), according to the manufacturer’s instruction. There was no serum collected for five participants.

### 4.4. Statistical Analyses

Differences between pre-intervention and post-intervention measures of variables were determined using paired t-test. Missing data are denoted in the text or by sample sizes in the tables and figure legends. Univariate regression analyses were used to determine relationships between ANGPTL4 expression and outcome variables. Statistical significance was set at *p* < 0.05. Data were analyzed by SPSS statistical software (Version 24, SPSS Inc., Chicago) and expressed as mean *±* SEM.

## 5. Conclusions

Our data indicate that skeletal muscle ANGPTL4 and PPARα exhibit differences by sex. ANGPTL4 expression in skeletal muscle is associated with serum ANGPTL4, glucose metabolism and obesity. Although six months of exercise training and weight loss did not change skeletal muscle AGNPTL4 in older adults, future investigations could explore the response of ANGPTL4 expression in adipose tissue.

## Figures and Tables

**Table 1:** Physical and metabolic characteristics and gene expression of all subjects (*n* = 35) before and after aerobic exercise + weight loss (AEX + WL) intervention.

Characteristic	Pre AEX + WL	Post AEX + WL
Weight (kg)	91.6 *±* 2.8	84.6 *±* 2.7 ^‡^
BMI (kg/m^2^)	31.3 *±* 0.7	28.5 *±* 0.7 ^‡^
Waist circumference (cm)	102.1 *±* 2.7	96.9 *±* 2.8 ^‡^
Hip circumference (cm) (*n* = 21)	114.9 *±* 2.5	110.4 *±* 2.2 ^‡^
Percent body fat	39.2 *±* 1.5	34.994 *±* 1.7 ^‡^
Fat mass (kg)	35.8 *±* 1.8	29.8 *±* 1.8 ^‡^
Fat-free mass (kg)	55.9 *±* 2.2	55.4 *±* 2.3
VO_2_max (mL/kg/min)	21.8 *±* 1.0	26.81 *±* 1.3 ^‡^
VO_2_max (L/min)	2.00 *±* 0.11	2.26 *±* 0.12 ^‡^
Fasting glucose (mmol/L)	5.41 *±* 0.10	5.25 *±* 0.09
120-min postprandial glucose (mmol/L)	8.82 *±* 0.4	7.35 *±* 0.37
Glucose_AUC_ (mmol/L/120 min)	980 *±* 37	926 *±* 36 ^†^
Glucose_AUC_ (mmol/L/180 min)	1260 *±* 51	1194 *±* 42 *
Fasting insulin (pmol/L)	87 *±* 7	69 *±* 5 ^‡^
Insulin_AUC_ (pmol/L/120 min)	53,098 *±* 4004	44,011 *±* 3178 ^‡^
Insulin_AUC_ (pmol/L/180 min)	67,294 *±* 5264	55,994 *±* 4316 ^‡^
Glucose utilization (mg/kg/min)	5.13 *±* 0.31	6.60 *±* 0.33 ^‡^
Glucose utilization (µmol/kg _FFM_/min)	48.01 *±* 3.29	56.50 *±* 2.86 ^‡^
Insulin sensitivity (mg/kg/min/min/pM)	0.026 *±* 0.002	0.036 *±* 0.002 ^‡^
Insulin sensitivity (µmol/kg _FFM_/min/pM)	0.044 *±* 0.004	0.054 *±* 0.003 ^‡^
Muscle ANGPTL4 expression (AU)	0.801 *±* 0.115	0.888 *±* 0.144
Muscle PPARα expression (AU) (*n* = 9)	16.15 *±* 2.163	15.62 *±* 2.469
Serum ANGPTL4 (pg/mL)	313.6 *±* 35.0	331.6 *±* 35.9
Visceral fat area (cm^2^)	160.1 *±* 14.7	127.5 *±* 12.5 ^‡^
Abdominal subcutaneous fat area (cm^2^)	356.6 *±* 19.0	301.2 *±* 21.3 ^‡^
Mid-thigh low density lean tissue area (cm^2^)	23.6 *±* 1.6	22.8 *±* 1.7
Mid-thigh muscle area (cm^2^)	90.4 *±* 5.8	94.7 *±* 5.0
Mid-thigh subcutaneous fat (cm^2^)	117.4 *±* 11.1	103.1 *±* 9.7
Mid-thigh muscle attenuation (HU) (n=15)	36.5 *±* 2.4	38.6 *±* 2.8 *
TG (mg/dL)	123 *±* 7	101 *±* 6
Total Cholesterol (mg/dL)	184 *±* 6	176 *±* 5 ^†^
HDL-Cholesterol (mg/dL)	44.7 *±* 1.8	47.5 *±* 1.8 ^†^
LDL-Cholesterol (mg/dL)	115.5 *±* 4.9	108.1 *±* 4.6 ^†^

Values are mean *±* SEM. Insulin and glucose area under the curve (AUC) from the oral glucose tolerance test (OGTT). Significant different pre and post intervention: * *p* ˂ 0.05; ^†^ *p* ˂ 0.01; ^‡^ *p* ≤ 0.001.

**Table 2:** Comparison of physical and metabolic characteristics and gene expression between men (*n* = 18) and women (*n* = 17) before the intervention.

Characteristic	Women	Men	*p* Value
Weight (kg)	82.7 *±* 4.0	100.1 *±* 2.9	0.010
BMI (kg/m^2^)	31.1 *±* 1.2	31.6 *±* 0.7	0.747
Percent body fat	45.6 *±* 1.4	33.1 *±* 1.3	0.000
Fat mass (kg)	38.6 *±* 3.0	33.7 *±* 2.0	0.194
FFM (kg)	44.7 *±* 1.3	67.2 *±* 1.7	0.000
VO_2_max (mL/kg/min)	19.8 *±* 1.2	24.2 *±* 1.4	0.022
Fasting glucose (mmol/L)	5.41 *±* 0.10	5.25 *±* 0.09	0.182
Fasting insulin (pmol/L)	87 *±* 13	87 *±* 7	0.953
Glucose utilization (mg/kg/min)	5.7 *±* 0.4	4.5 *±* 0.4 (*n* = 15)	0.051
Glucose utilization (µmol/kg _FFM_/min)	58.3 *±* 4.2	36.4 *±* 3.1 (*n* = 15)	0.000
Insulin sensitivity (mg/kg/min/min/pM)	0.029 *±* 0.003	0.021 ± 0.002 (*n* = 13)	0.048
Insulin sensitivity (µmol/kg _FFM_/min/pM)	0.053 *±* 0.005	0.032 *±* 0.003 (*n* = 13)	0.001
TG (mg/dL)	127 *±* 9	120 *±* 11	0.607
Total Cholesterol (mg/dL)	201 *±* 9	169 *±* 6	0.005
HDL-cholesterol (mg/dL)	50.1 *±* 2.8	39.7 *±* 1.5	0.003
LDL-cholesterol (mg/dL)	125.9 *±* 7.6	105.6 *±* 5.3	0.038
Muscle ANGPTL4 expression (AU)	1.174 *±* 0.167	0.507 *±* 0.119	0.003
Muscle PPARα expression (AU)	10.60 *±* 1.353 (*n* = 9)	20.46 *±* 3.036 (*n* = 9)	0.013
Serum ANGPTL4 (pg/mL)	374.3 *±* 71.4 (*n* = 15)	309.2 *±* 31.5 (*n* = 15)	0.415

Values are mean *±* SEM.

**Table 3:** Relationship between skeletal muscle ANGPTL4 mRNA and metabolic variables.

All Participants	r	*p*
Percent body fat (*n* = 35)	0.64	0.000
Fat mass (*n* = 35)	0.48	0.004
Fat-free mass (*n* = 35)	−0.45	0.007
Mid-thigh muscle area (*n* = 33)	−0.56	0.001
Mid-thigh subcutaneous fat area (*n* = 33)	0.52	0.002
Visceral fat area (*n* = 34)	0.23	0.196
Total cholesterol (*n* = 35)	0.43	0.010
HDL-cholesterol (*n* = 35)	0.35	0.037
LDL-cholesterol (*n* = 35)	0.37	0.027
Triglyceride (*n* = 35)	0.07	0.717
VO_2_max (mL/kg/min) (*n* = 35)	−0.57	0.001
M (µmol/kg _FFM_/min) (*n* = 32)	0.38	0.033
Glucose_AUC_ (mmol/L/120 min) (*n* = 34)	−0.01	0.965
Glucose_AUC_ (mmol/L/180 min) (*n* = 34)	0.12	0.500
Insulin_AUC_ (pmol/L/120 min) (*n* = 34)	0.28	0.115
Insulin_AUC_ (pmol/L/180 min) (*n* = 33)	0.18	0.327
Serum ANGPTL4 (*n* = 30)	0.41	0.025
